# Interpretation of surficial shear crack propagation mechanisms in bending for Zn or AlSi coated hot press forming steels

**DOI:** 10.1038/s41598-021-91065-x

**Published:** 2021-06-01

**Authors:** Selim Kim, Min Cheol Jo, Seongwoo Kim, Jinkeun Oh, Sang-Heon Kim, Seok Su Sohn, Sunghak Lee

**Affiliations:** 1 Center for Advanced Aerospace Materials, Pohang University of Science and Technology, Pohang, 37673 Republic of Korea; 2 Automotive Steel Research Group, Technical Research Laboratories, POSCO, Kwangyang, 57807 Republic of Korea; 3 Department of Materials Science and Engineering, Korea University, Seoul, 02841 Republic of Korea

**Keywords:** Mechanical properties, Metals and alloys

## Abstract

The bending angle at the peak load is regarded as the most important parameter for evaluating bending properties of hot-press-forming (HPF) steels. However, it is not a mechanics-based parameter for the bending criterion, and the data interpretation is difficult because bending criteria in relation with microstructures and associated bending mechanisms have not been verified yet. In this study, effects of coating and baking treatments on bending angles at the peak load of three kinds of 1470 MPa-grade HPF steels were investigated by interrupted three-point bending tests coupled with direct microstructural observation. According to direct observations of sequential cracking processes of V-shaped crack (V-crack), bending procedures were classified into four stages: (1) formation of small V-crack, (2) increase in number and size of V-cracks, (3) initiation of shear-crack propagation from the V-crack tip, and (4) further propagation and opening of the shear crack. The minimum bending angle required for initiating the shear-crack propagation from the V-crack tip was defined as a critical angle, which meant the boundary between the 2nd and 3rd stages. The present bending behavior related with critical bending angle and V-cracking could be interpreted similarly by the fracture-mechanics concept, i.e., the initiation of shear-crack propagation.

## Introduction

Giga-grade high-strength hot-press-forming (HPF) steel sheets have a martensitic structure obtained from high-temperature austenitization, press-forming, and rapid-cooling processes to meet the strength requirements for automotive reinforcement components^1–5^. To achieve the giga-grade strength, however, often deteriorates bending properties related with sheet formability, which limits their wide applications in automotive industries. Thus, the enhancement of bending properties has been actively investigated by changing a coating process^6,7^, applying a baking treatment^8,9^, reducing a carbon content^10–12^, and refining prior austenite grains^13–15^.

The bending angle at the peak load is regarded as the most important parameter for evaluating bending properties in laboratorial scale three-point bending tests which correspond to the bending behavior of industrial scale HPF steel sheets. However, it is not a mechanics-based parameter for the bending criterion, and the data interpretation might be difficult because detailed bending mechanisms were not clarified yet. In order to quantitatively evaluate the bending parameters, therefore, intensive studies on bending criteria in relation with microstructures and associated bending mechanisms are required.

In the present study, effects of coating and baking treatments on bending angle at the peak load were investigated in high-strength HPF steel sheets via interrupted three-point bending tests coupled with detailed microstructural examination. Bending deformation mechanisms were sequentially analyzed by morphological changes of V-shaped cracks formed at the outer surface of the bend specimen on which large tensile strains were applied. A critical bending angle required for initiating the shear-crack propagation was defined for quantitatively evaluating the critical bending parameters.

## Results

### Microstructures and hardness values of three HPF steels

**Figure 1:**
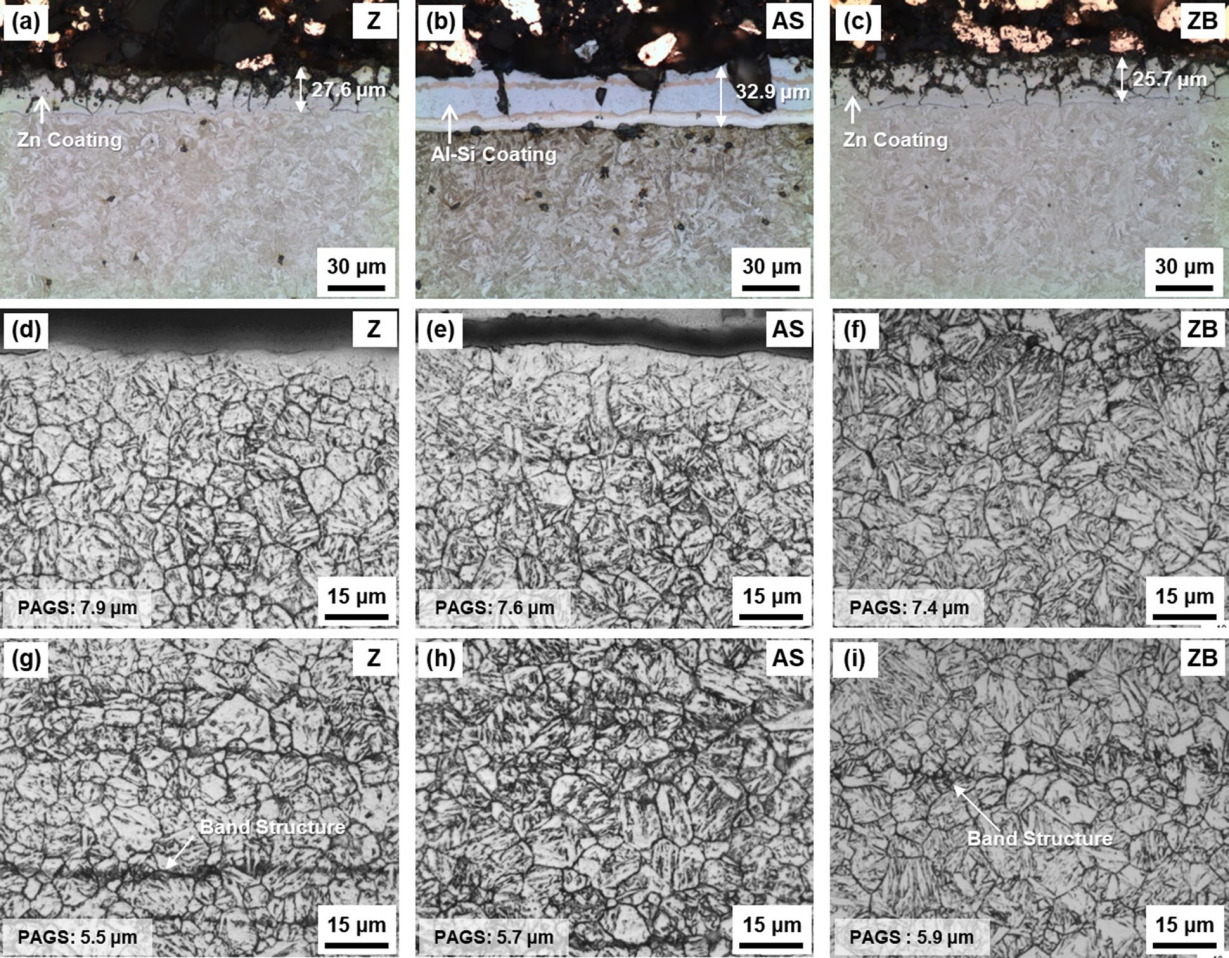
Optical micrographs of the (**a**–**c**) surface, (**d**–**f**) coating/steel interface, and (**g**–**i**) interior matrix areas of the three HPF (Z, AS, and ZB) steels. The average thickness of coating layers of the Z, AS, and ZB steels is 27.6, 32.9, and 25.7 μm, respectively. All the steels are composed of full martensite.

**Figure 2:**
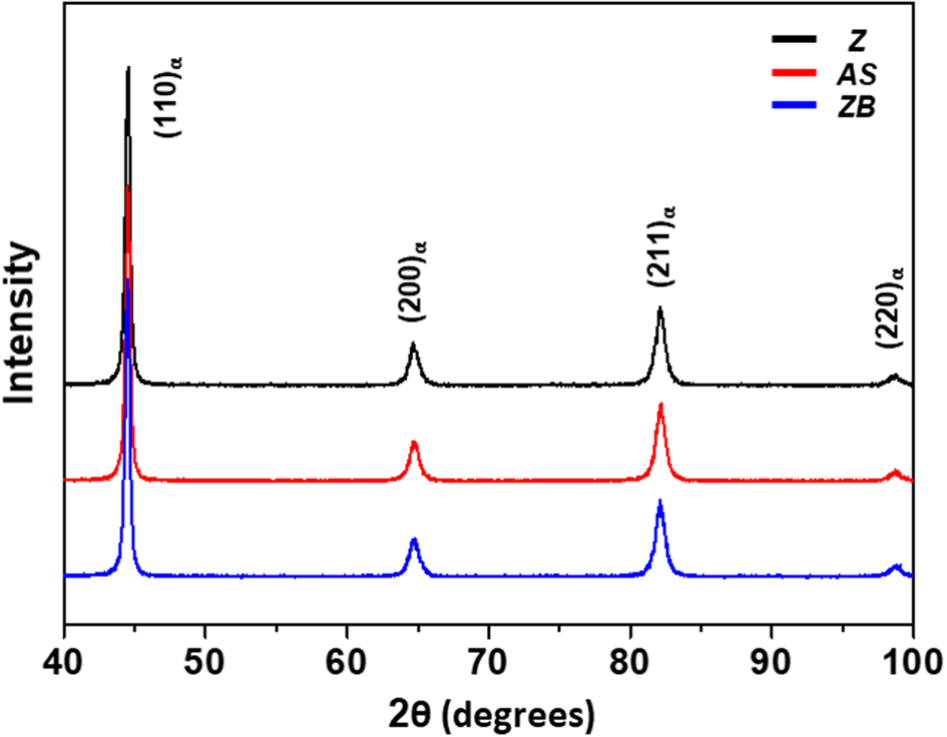
X-ray diffraction patterns of the Z, AS, and ZB steels, showing peaks of body-centered-cubic (BCC) phase.

Figure 1a–c shows optical micrographs of the surface area of the three HPF (Z, AS, and ZB) steels. The average thickness of coating layers of the Z, AS, and ZB steels is 27.6, 32.9, and 25.7 μm, respectively. Figure 1d–i shows optical micrographs of the coating/steel interfacial and interior matrix areas, respectively. All the steels are composed of full martensite (or tempered martensite) irrespective of interfacial or interior area, and some band structures are slightly developed. XRD profiles in Fig. 2 indicate only BCC-structured peaks present in three steels. Full width at half maximum (FWHM) for (110)_α_ peak was measured to be 0.36, 0.38, and 0.38 degrees for Z, AS, and ZB steels, respectively, showing there is no dislocation density reduction at least according to the peak broadening of the XRD profiles. The bands generally form by the segregation of substitutional alloying elements like Mn, Ni, and Mo during the casting of conventional high-alloyed steels, and they are hardly removed by the practical homogenization^16^. It is not likely that the bands influence the bendability because they are weakly formed and their existence is generally approved in steel industries. As prior austenite grain boundaries are clearly revealed by the etching, the measured prior austenite grain sizes (PAGSs) are indicated inside the micrographs. The PAGSs of the interfacial and interior areas are 7.2 and 5.5 μm, respectively, in the Z steel, and are also similar in the AS and ZB steels. This result indicates that the baking at 443 K in the ZB steel hardly affects the overall martensitic microstructure. The Vickers hardness of the interfacial and interior areas was measured, and the data are summarized in Table 1, together with the coating thickness and PAGS. In the three steels, the hardness of interfacial area is higher than that of the interior area. It decreases in the order of the AS, Z, and ZB steels, while the hardness of interior area is similar in the three steels.

**Table 1:** Coating thickness, prior austenite grain size (PAGS), and Vickers hardness of the Z, AS, and ZB steels.

Steel	Coating thickness (μm)	PAGS (μm)		Vickers hardness (VHN)	
		Interface	Interior	Interface	Interior
Z	27.6 ± 2.0	7.9 ± 1.0	5.5 ± 0.6	512.5 ± 10.6	481.1 ± 11.1
AS	32.9 ± 2.6	7.6 ± 0.9	5.7 ± 0.9	528.7 ± 12.5	483.3 ± 8.3
ZB	25.7 ± 1.5	7.4 ± 0.8	5.9 ± 0.8	503.5 ± 12.9	477.3 ± 9.6

**Figure 3:**
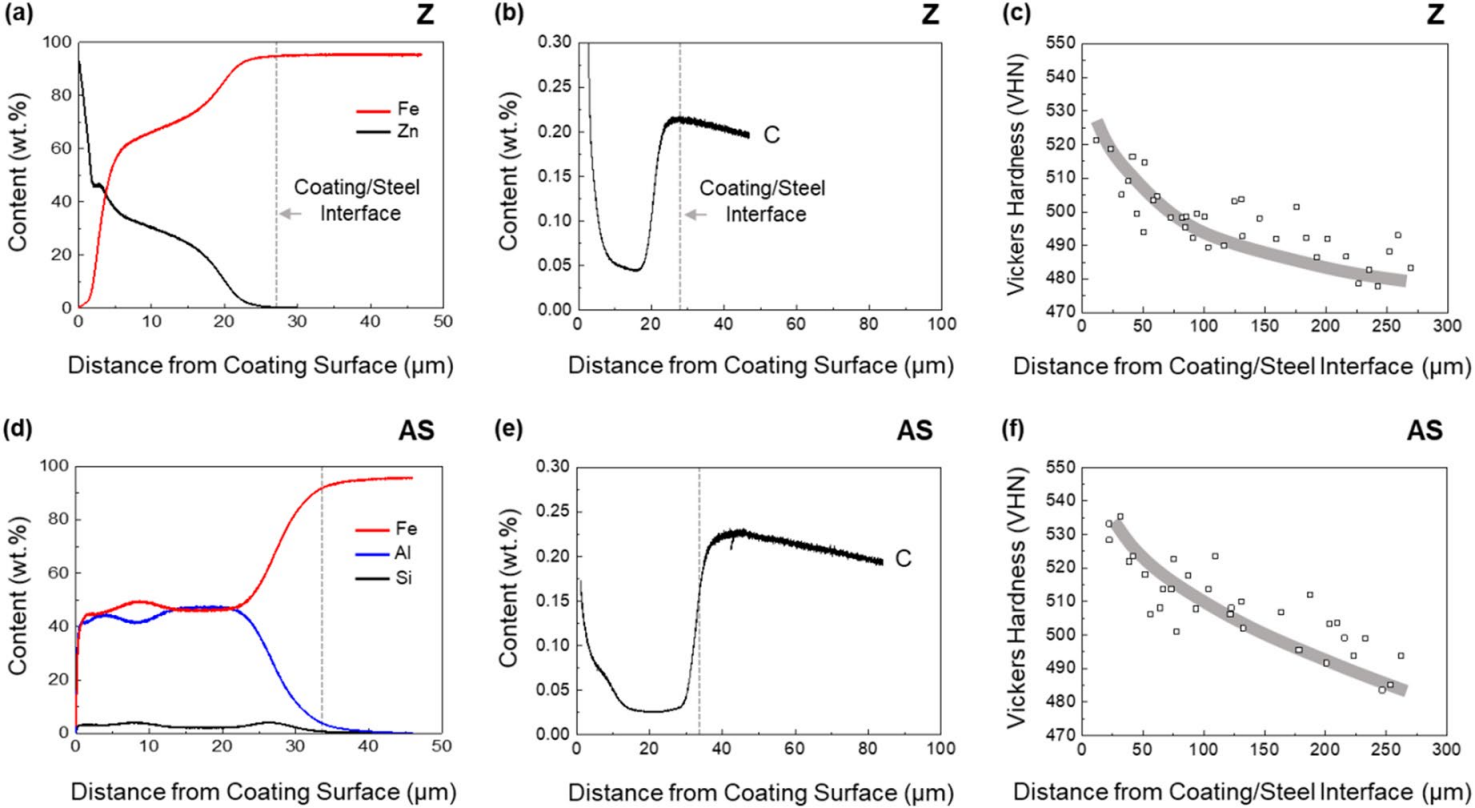
(**a**,**b**,**d**,**e**) Glow discharge spectrometer (GDS) data of Fe, Zn, Al, Si, and C contents as a function of distance from the coating surface in the Z and AS steels. (**c**,**f**) Shows Vickers hardness data (indentation load; 100 g) from the interface to the interior.

Figure 3a,b,d,e shows the glow discharge spectrometer (GDS) data of Fe, Zn, Al, Si, and C contents as a function of distance from the coating surface in the Z and AS steels. As the detecting point moves from the coating surface to the coating/steel interface, the content of Zn decreases continuously (Fig. 3a), while those of Al and Si almost remain near the coating surface and then decrease relatively rapidly near the interface (Fig. 3d). Though the C content is low at a 0.05 wt% level in the coating, it is about 0.22 and 0.24 wt% near the coating/steel interfacial area of the Z and AS steels, respectively. This C concentration is introduced from the steel substrate during the coating process. A diffusion layer is generated on the substrate surface area by a reaction of the Zn or Al-Si coating layer and the substrate. According to Fan et. al.^17^, the melting point of an aluminized coating was 577 °C, and Al and Si were dissolved to form intermetallic phases such as FeAl, Fe_2_Al_5,_ and Fe_2_SiAl_7_ during the high-temperature HPF procedures for several minutes. In the present coated steel sheet, FCC-base austenite has the high C solubility (< 2%) as the C content is determined during austenitizing at 900 °C for 6 min followed by water quenching. Since the C solubility of Fe–Al-base intermetallic phases is very low^18^, the undissolved C is accumulated at the steel substrate side of the coating/steel interface. Janik et al.^19^ reported in Zn-coated steel sheets that Zn–Fe intermetallic phases were transformed to a Zn-rich ferrite after the HPF procedure at 900 °C for 3 min as they were not thermodynamically stable. Thus, the C content reaches the peak near the coating/steel interface in the AS and Z steels, as shown in Fig. 3b,e. As a result, the carbon enrichment occurs at the local substrate surface, i.e., coating/steel interface.

For the AS steel having the thicker coating layer, the GDS experiment cannot show the C profile enough to reach the steel substrate due to the limited analysis depth (~ 50 μm). Thus, the further experiment was performed again after removing the surface layer. The C content decreases in both the Z and AS steels as the detecting point moves from the interface to the interior, and overall C content is higher in the AS steel (0.21–0.24 wt%) than in the Z steel (0.19–0.21 wt%) at the similar position.

Figure 3c,f shows the Vickers hardness data (indentation load: 100 g) from the interface to the interior. In the Z steel, the Vickers hardness is about 515 ± 6.2 VHN at the interface and continuously decreases to reach the matrix hardness of about 484 ± 5.0 VHN (Fig. 3c). Though the hardness trend of the AS steel is similar to that of the Z steel, the hardness at the interface of the AS steel is lower than that of the Z steel as shown in Table 1 and Fig. 3f.

### Tensile and bending properties of three HPF steels

**Figure 4:**
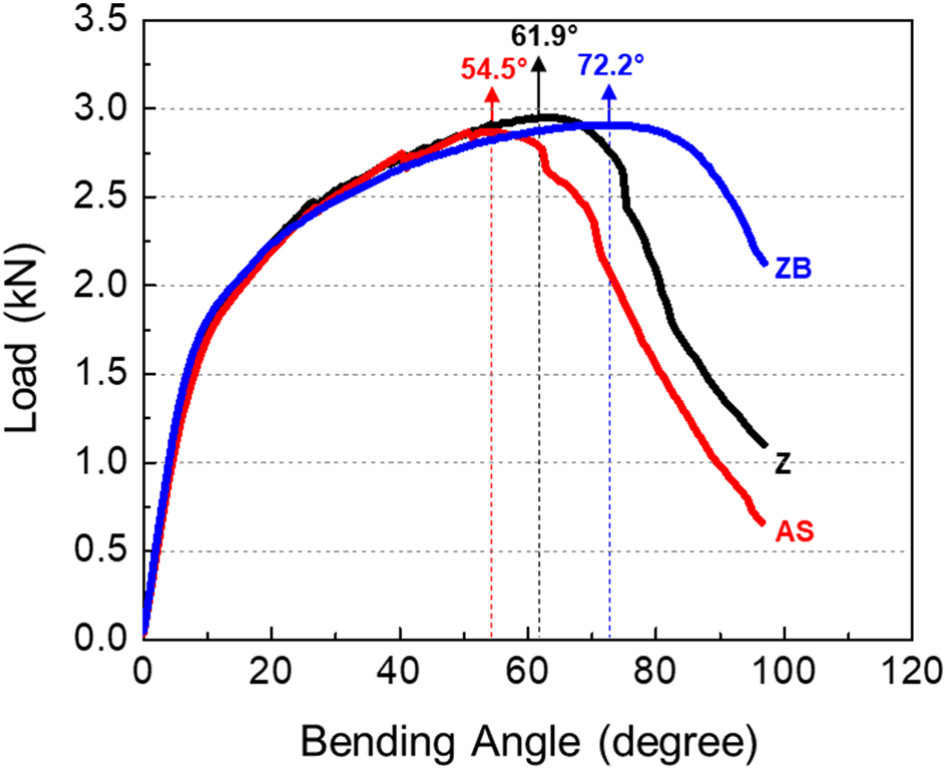
Load-bending-angle curves of the Z, AS, and ZB steels. The bending angle at the peak load is lowest in the AS steel, and increases in the order of the Z and ZB steels.

Room-temperature tensile properties of the Z, AS, and ZB steels are shown in Table 2. Overall properties are similar in the Z and AS steels. After the baking of the Z steel, the yield strength increases, while the tensile strength decreases slightly, and the elongation is similar. Figure 4 shows load-bending-angle curves of the three steels, and the data are summarized in Table 2. Since all the present bending tests were performed after about 3 months at least from commercial HPF procedures, it is acceptable that their results are not affected by the amount of H existed in the steel specimens under the H-uptake HPF environment^20^. The curve shape is similar in the three steels. The bending angle at the peak load (peak-load bending angle) is lowest in the AS steel, and increases in the order of the Z and ZB steels as indicated by arrow marks in Fig. 4.

**Table 2:** Room-temperature tensile and bending test results of the Z, AS, and ZB steels.

Steel	Yield strength (MPa)	Tensile strength (MPa)	Elongation (%)	Peak-load bending angle (°)
Z	1112 ± 4	1487 ± 1	6.1 ± 0.1	61.9 ± 1.13
AS	1093 ± 10	1519 ± 3	6.7 ± 0.3	54.5 ± 0.96
ZB	1201 ± 8	1452 ± 9	6.1 ± 0.2	72.2 ± 1.48

### Direct observation of bending procedures: V-crack formation

**Figure 5:**
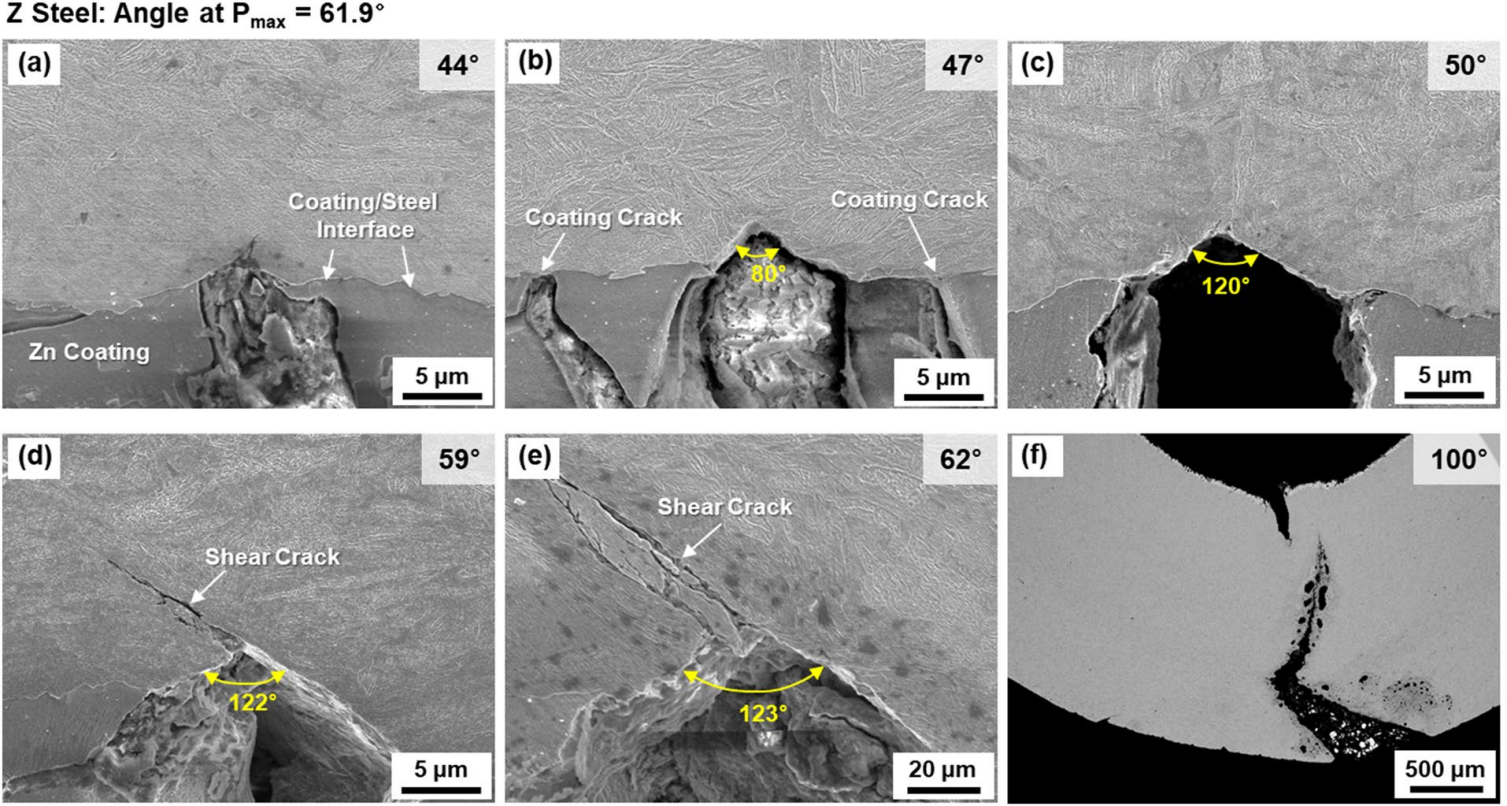
(**a**–**f**) SEM micrographs illustrating sequential V-cracking processes at various bending angles in the Z steel. Bending procedures are classified into four stages: (**a**,**b**) formation of small V-crack, (**c**) increase in number and size of V-cracks, (**d**) initiation of shear-crack propagation from the V-crack tip, and (**e**,**f**) further propagation and opening of shear crack to reach the failure.

To investigate effects of bending mechanisms at the tensile-strained area (central area of the outer surface) on peak-load bending angles, interrupted bending tests were carried out. Sequential cracking processes at various angles in the Z steel are shown in Fig. 5a–f. There exist no cracks or grooves at the angle of 44°, while a short micro-crack is found at the coating/steel interface (Fig. 5a). At 47°, a small V-shaped crack (referred to as ‘V-crack’ for convenience) forms, and the angle measured at the V-crack tip is 80° as shown in Fig. 5b. Cracks form in the coating, but do not penetrate into the steel interior. At 50° (Fig. 5c), the overall size and tip angle of the V-cracks tend to slightly increase. Two V-cracks form at this stage, but Fig. 5c shows only one of them representatively. While the angle increases until 59°, the number and size of V-cracks increase steadily. At 59°, the V-crack tends to enlarge further, and a shear crack starts to propagate from the V-crack tip along the shear direction (about 45° direction to the tensile loading direction) (Fig. 5d). At 62° which is the peak-load angle (Fig. 4), the crack enlarges and propagates further into the interior (Fig. 5e). At 100°, the propagated crack is largely opened, while another crack propagates from the inner specimen surface (Fig. 5f), and the bending load decreases as the cross-sectional area of the specimen reduces. According to these observations of V-crack, bending procedures are classified into four stages: (1) formation of small V-crack (Fig. 5a,b), (2) increase in number and size of V-crack (Fig. 5c), (3) initiation of shear-crack propagation from the V-crack tip (Fig. 5d), and (4) further propagation and opening of shear crack to reach the failure (Fig. 5e,f).

**Figure 6:**
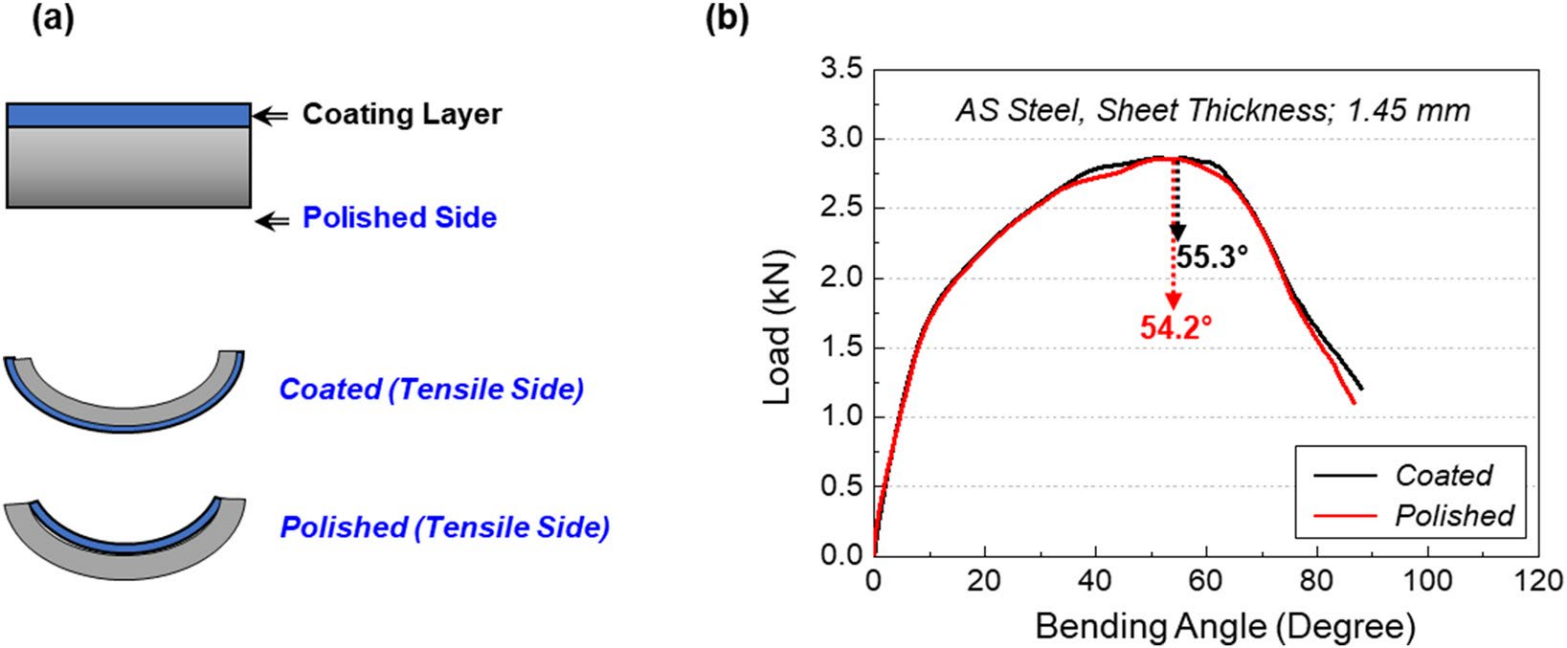
(**a**) Schematic diagrams of shapes of the bending specimen where the coating or polished-substrate surface is tensile-strained during the bending test, which is referred to as ‘Coated’ or ‘Polished’, respectively. (**b**) Load-bending-angle curves of the Coated and Polished bending specimens for the AS steel.

In order to confirm the effect of the coating on peak-load bending angle, bending tests of the AS steel were conducted after one side of coated surfaces was polished out. Figure 6a shows schematic diagrams of shapes of the bending specimen where the coating or polished-substrate surface is tensile-strained during the bending test, which is referred to as ‘Coated’ or ‘Polished’, respectively. Figure 6b shows load-bending-angle curves of the Coated and Polished bending specimens for the AS steel, and the peak-load angles are indicated by dashed arrows. The peak-load angle of the Coated bending specimen is almost similar to that of the Polished specimen (55.3° vs. 54.2°). This indicates that the presence of the coating does not play a role in deteriorating the peak-load angle because cracks formed in the coating do not penetrate into the steel interior to deteriorate the bendability.

**Figure 7:**
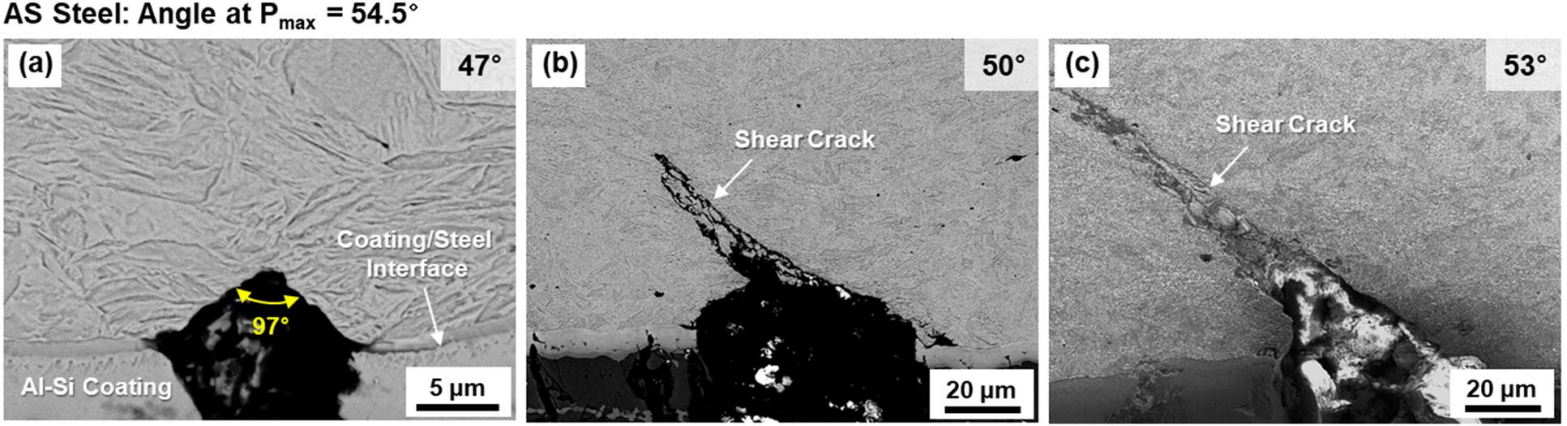
(**a**–**c**) SEM micrographs illustrating sequential V-cracking processes at various bending angles in the AS steel. The second bending stage (increase in number and size of V-cracks) does not occur as the shear crack initiated at the V-crack tip propagates rapidly without enlarging the V-crack or forming other V-cracks.

Figure 7a–c shows series of SEM micrographs of V-cracking behavior of the AS steel. At the angle of 47°, a small V-crack whose tip angle is 97° appears (Fig. 7(a)). At 50°, there exists a wide V-crack (tip angle; 112°), from which a shear crack propagates along the 45° direction (Fig. 7b). Here again, a few cracks form in the coating, but do not affect the V-crack formation. At 53° [close to the peak-load angle (Fig. 4)], a shear crack propagates further from the V-crack tip (Fig. 7c). When these observations are compared with those of the Z steel, the second bending stage (increase in number and size of V-cracks) does not occur as the shear crack initiated at the V-crack tip propagates rapidly without enlarging the V-crack or forming other V-cracks.

**Figure 8:**
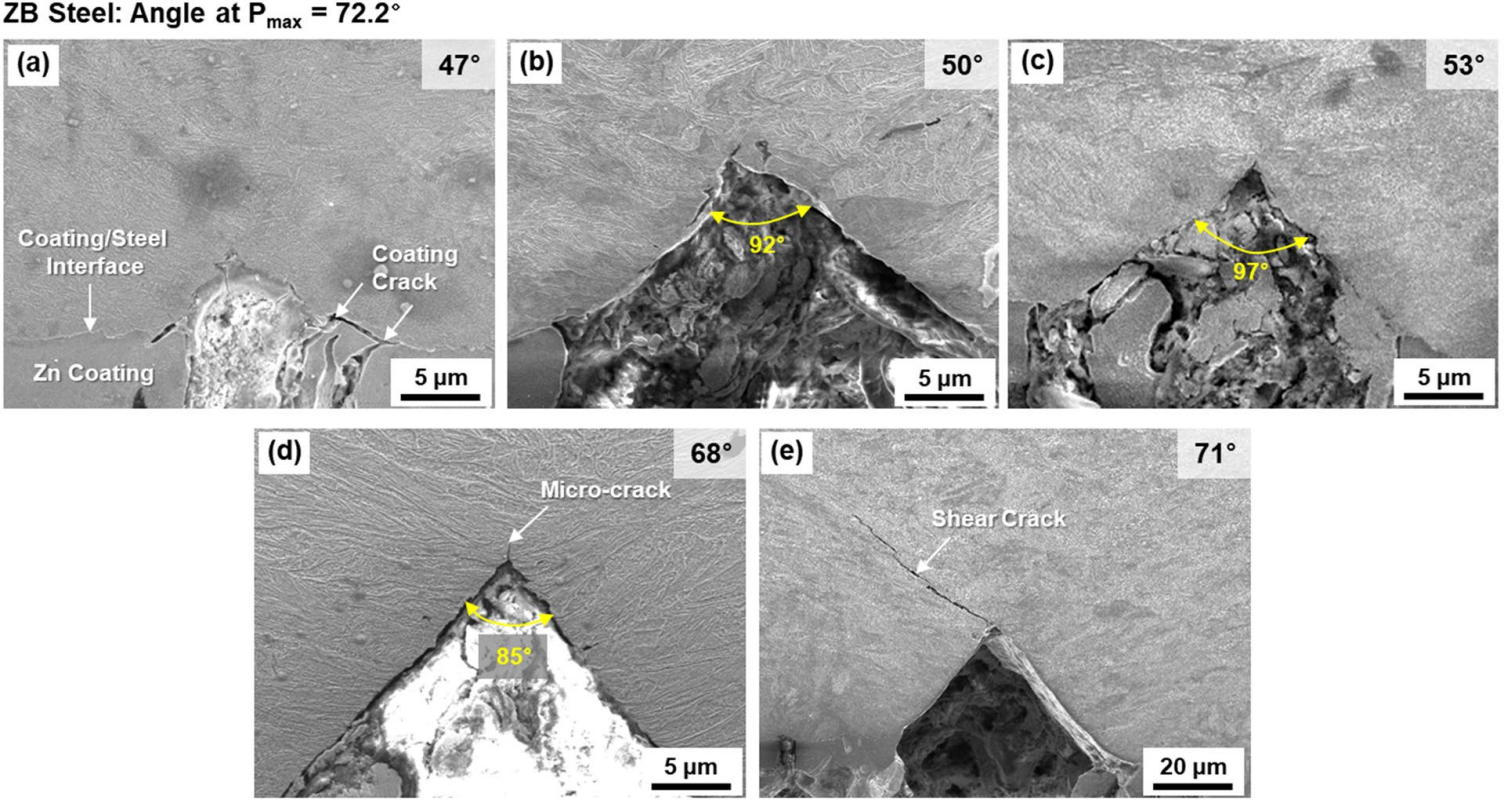
(**a**–**e**) SEM micrographs illustrating sequential V-cracking processes at various bending angles in the ZB steel.

Figure 8a–e shows the sequential V-cracking behavior in the ZB steel. At 47°, there is a small U-shaped groove which might act as a precursor of V-cracking (Fig. 8a). At 50°, an enlarged V-crack starts to form (Fig. 8b). At the higher bending-angle range of 53°–66°, many V-cracks form, and their sizes or angles tend to increase with increasing bending angle (Fig. 8c). At 68°, a V-crack enlarges further, and a micro-crack starts to propagate at the V-crack tip (Fig. 8d). At 71° (peak-load angle) (Fig. 4), a shear crack propagates further from the V-crack tip (Fig. 8e).

## Discussion

### Definition of critical bending angle for initiation of shear-crack propagation

**Figure 9:**
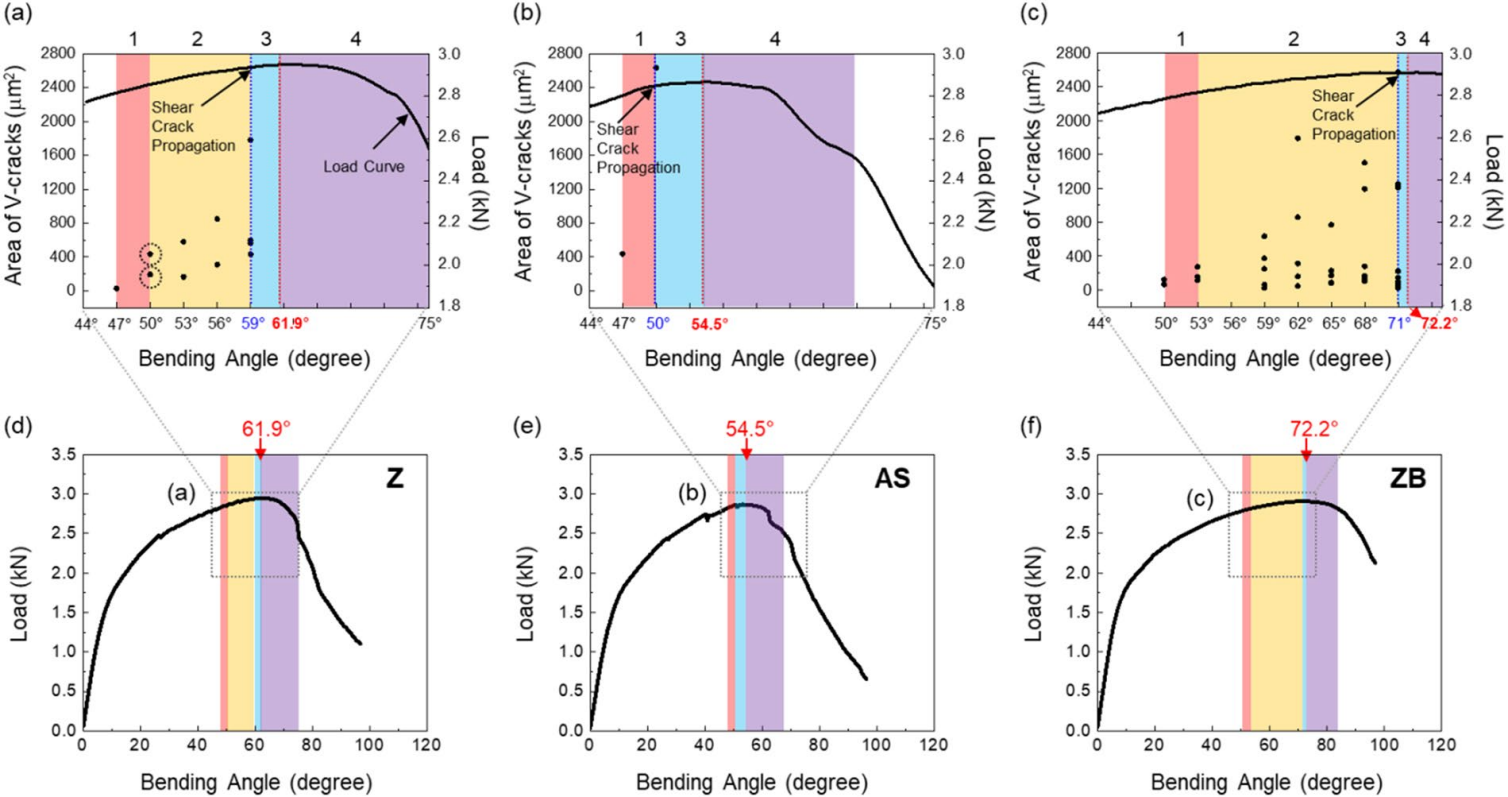
(**a**–**c**) Area and number of V-cracks and (**d**–**f**) bending load as a function of bending angle for the Z, AS, and ZB steels. The bending angle range (44°–75°) of the dashed-line boxes of (**d**–**f**) is shown in (**a**–**c**). The four bending stages are indicated respectively by orange, yellow, light-blue, light-purple colors in these figures.

Variations in bending angle and associated bending mechanisms can be interpreted by main fracture-initiation factors, i.e., V-cracking and shear-crack propagation at the central area of the outer specimen surface. The width, depth, crack-tip angle, and area of V-crack can be listed as microstructural parameters for evaluating the sequential V-cracking process. Among them, the crack area is selected in this study as a main evaluating parameter because it represents well the overall V-cracking behavior, and is plotted as a function of bending angle in Fig. 9a–c for the Z, AS, and ZB steels. These plots are also matched with the load-bending-angle curves of Fig. 4, as shown in Fig. 9d–f. Here, the angle range (44°–75°) of the dashed-line boxes of Fig. 9d–f is expanded in Fig. 9a–c.

In the Z steel, a V-crack starts to form at the angle of 47° (1st cracking stage), and two V-cracks having the larger crack area form at 50° (2nd stage), as shown in Fig. 9a. Here, two black-dot symbols (dotted-circle marks) mean that the crack areas of the two cracks are 192 and 434 μm^2^. The number and area of V-cracks tend to increase as the 2nd stage continues from 50°–59°. At 59°, four V-cracks form, and a shear crack starts to propagate at one of V-crack tips (3rd stage). At the higher angles, the shear crack propagates further to reach the failure (4th stage). According to these categories, the four bending stages are defined, as indicated respectively by orange, yellow, light-blue, light-purple colors in Fig. 9a,d.

In the AS steel, a small V-crack forms at 47°, which is same to the angle for the Z steel, in the 1st stage (Fig. 9b). At 50°, a V-crack forms, enlarges, and then propagates rapidly without the 2nd stage, as indicated by orange, light-blue, and light-purple colors. This AS steel presents a brittle fracture behavior showing the rapid initiation and propagation of V-crack. The initiation and propagation procedures of V-cracks are slowed down in the ZB steel. A small U-crack appears at 47°, and develops into a V-crack at 50° in the 1st stage (Fig. 9c). Until 68°, many V-cracks form and enlarge as indicated by the wide yellow-colored region, and a shear crack finally starts to propagate from a V-crack tip at 71°, which shows the end of the 2nd stage.

The minimum bending angle required for initiating the shear-crack propagation from the V-crack tip is defined as a critical angle, which means a boundary between the 2nd and 3rd stages (between yellow- and light-blue-colored ranges in Fig. 9a–f). The critical bending angle is 59°, 50°, and 71° in the Z, AS, and ZB steels, respectively. The difference in critical angle between the Z and AS steels is attributed mainly to the hardness of the coating/steel interfacial area of the outer surface. In other words, the harder interfacial area of the AS steel (Fig. 3d and Table 1) results in the easy initiation of shear-crack propagation right after the V-crack formation because the V-crack formed at the harder area can readily provide the crack propagation path at its tip (Fig. 7b). The reason for the different critical angle between the Z and ZB steels cannot be easily explained by the hardness difference because the basic structure and hardness at the interfacial area are not much different (Fig. 1d,f and Table 1). Small microstructural changes after the low-temperature baking, such as dissolution of retained austenite, or precipitation of ε-carbides^8,21–24^, might exist, but their verification is also difficult in the martensitic HPF steels^3,9^.

Cho et al.^9^ reported that the hydrogen content existed inside an AlSi-coated 1900 MPa-grade HPF steel increased from 0.055 to 0.502 ppm after the HPF process, which resulted in the serious reduction in a peak-load angle. After the baking at 443** °C**, however, the hydrogen content reduced to 0.369 ppm, thereby leading to the enhancement in bending angle. In the present Zn-coated and baked ZB steel, thus, the great enhancement in critical bending angle from 59°–71° might result from the reduction in hydrogen content after the baking, although the detailed hydrogen charging and measurement were not carried out in this study.

### Critical bending angle and peak-load bending angle

**Figure 10:**
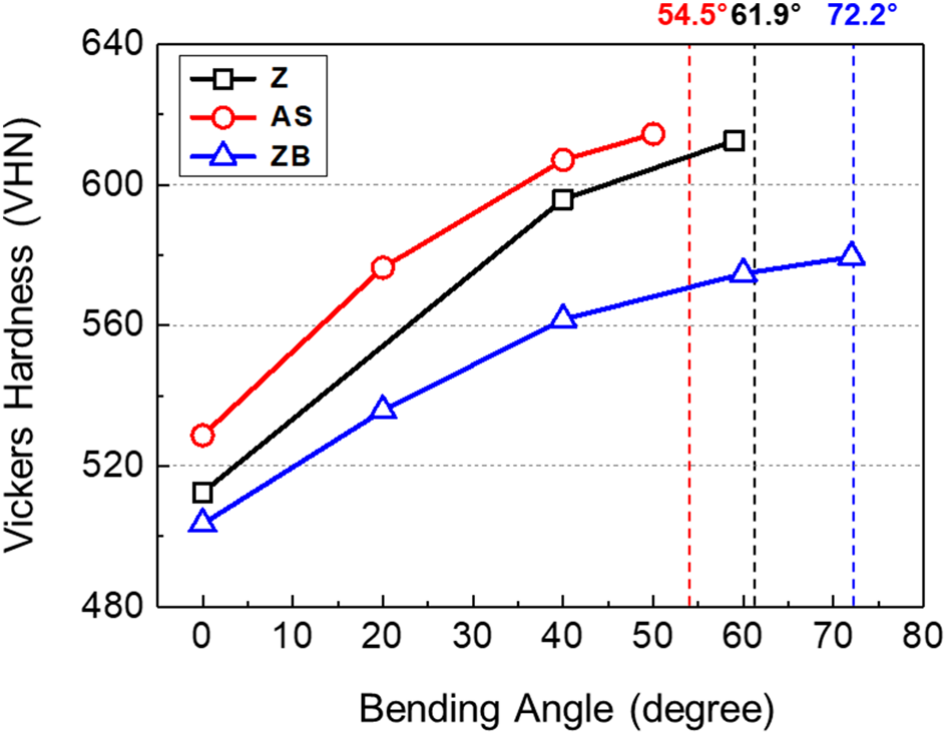
Vickers hardness of the coating/steel interfacial area as a function of bending angle for the Z, AS, and ZB steels. The hardness increases almost linearly with increasing bending angle in the three steels.

It is interesting to note that the critical angles required for the initiation of shear-crack propagation (59°, 50°, and 71° in the Z, AS, and ZB steels, respectively) are smaller by 1°–4° than the peak-load angles (62°, 54°, and 72°). This implies that the peak-load point lies right after the critical angle point. In order to explain the difference between the two angles, the hardness of the interfacial area was measured until the peak-load angle, and the results are shown in Fig. 10. The hardness increases almost linearly with increasing bending angle in the three steels. The bending load increases a little bit further after the critical angle, as indicated by the light-blue-colored region in Fig. 9a–c, although the shear-crack propagation reduces the cross-sectional area of the bending specimen. This indicates that the strain hardening due to the bending deformation at the interfacial area retards the drop of bending load. Thus, the peak-load angle is slightly larger (by 1°–4°) than the critical angle for initiation of shear-crack propagation. It is also noted that the slope between hardness and bending angle, which implies a kind of strain hardening rate, is 1.72, 1.67, and 1.06 VHN/deg in the AS, Z, and ZB steels, respectively. The trend of the slope is same to that of the gap between the peak-load angle and critical angle, i.e., 4.5°, 2.9°, and 1.2°, in the AS, Z, and ZB steels, respectively, as indicated by light-blue-colored regions in Fig. 9a–f. This result indicates that the degree of the retardation of bending load drop is closely related with the strain hardening rate due to the bending deformation because it increases in the order of the ZB, Z, and AS steels.

Another noteworthy bending phenomenon is the similar or same angle for V-crack formation in the 1st stage, i.e., 47°, 47°, and 50° in the Z, AS, and ZB steels, respectively. This is because the three steels have the basically almost same martensitic matrix (Fig. 1g–i), while the microstructures of the interfacial area are varied by the coating or baking treatment (Fig. 1d–f). This also indicates that the microstructures of the interior and interfacial areas influence the bending angles at the starting point of the 1st and 3rd stages, respectively. Therefore, the angle for V-crack formation in the 1st stage is closely related to the interior area composed of the unvaried martensitic matrix, while the critical angle for initiation of shear-crack propagation depends on the interfacial area which is varied by the coating or baking treatment.

This study on critical bending-angle concept in the 1470 MPa-grade HPF steels gives a promising method to investigate the correlation of V-crack formation and bending properties and to understand the fracture-mechanics concept of V-cracks. Bending properties of the coated or baked HPF steels can be explained by sequential four bending stages, i.e., (1) small V-crack formation, (2) V-crack population, (3) initiation of shear-crack propagation, and (4) further crack propagation, in relation to the major fracture-initiating parameter, i.e., critical bending angle. Since the quantitative microstructural and mechanical analyses of V-cracks formed in the bending specimens have been hardly carried out because of their microstructural complexity and small size, the present interrupted bending test is outstanding for the microstructure- and mechanics-focused interpretation of bending test data.

The present bending behavior related with the critical bending angle and V-cracking in the high-strength HPF steels can be interpreted similarly by the fracture-mechanics concept, i.e., the blunting and initiation of pre-fatigued crack propagation in conventional high-strength steels^25–28^. The plane-strain fracture toughness is defined as the plane-strain value of critical stress intensity factor at the initiation of crack propagation after the sharp-crack-tip blunting^29–33^. Like this fracture toughness definition, the peak-load bending angle can be defined as the critical angle for initiation of shear-crack propagation, although there exists a small gap (1°–4°) between the two angles because of the strain-hardening effect at the V-crack-tip area (Fig. 10). Thus, it is expected that the idea of critical angle for initiation of shear-crack propagation can also be applied to strong and tough martensitic (or tempered martensitic) steels as well as stronger 1.9-GPa-grade HPF steels requiring tensile properties, toughness, and bendability.

## Conclusions

Effects of coating and baking treatments on critical or peak-load bending angles of three 1470 MPa-grade HPF steels were investigated by interrupted three-point bending tests coupled with direct microstructural examination.

1. The three steels were composed of full martensite, whose prior austenite grain sizes of the coating/steel interfacial and interior areas were about 7 and 5 μm, respectively. This indicated that the baking at 170 °C hardly affected the overall martensitic microstructure, which could be confirmed due to the hardness of interior area was hardly varied. However, the hardness of interfacial area was lower than that of the interior area, and decreased in the order of the AS, Z, and ZB steels.
2. According to direct observations of sequential V-cracking processes of the Z steel, bending procedures were classified into four stages: (1) formation of small V-crack, (2) increase in number and size of V-cracks, (3) initiation of shear-crack propagation from the V-crack tip, and (4) further propagation and opening of shear crack to reach the failure. The AS steel displayed a brittle fracture behavior showing the rapid initiation and propagation of V-crack, whereas the initiation and propagation procedures of V-cracks were slowed down in the ZB steel.
3. The minimum bending angle required for initiating the shear-crack propagation from the V-crack tip was defined as a critical angle, which meant the boundary between the 2nd and 3rd stages, and was 59°, 50°, and 71° in the Z, AS, and ZB steels, respectively. The difference in critical angle between the Z and AS steels (59° vs. 50°) was attributed mainly to the hardness of the coating/steel interfacial area. The different critical angle between the Z and ZB steels (59° vs. 71°) might result from the reduction in hydrogen content after the baking.
4. The microstructures of the interior and coating/steel interfacial areas influenced the bending angles in the 1st and 3rd stages, respectively. That is, the angle for V-crack formation in the 1st stage was closely related to the interior area composed of the unvaried martensitic matrix, while the critical angle for initiation of shear-crack propagation depended on the interfacial area which was varied by the coating or baking treatment.
5. The present bending behavior related with critical bending angle and V-cracking could be interpreted similarly by the fracture-mechanics concept, i.e., the blunting and initiation of pre-fatigued crack propagation. Like the definition of plane-strain fracture toughness, the peak-load bending angle, which was the most important parameter for evaluating bending properties, could be defined as the critical angle for initiation of shear-crack propagation, although there existed a small gap (1°–4°) between the two angles because of the strain-hardening effect at the V-crack-tip area.

## Methods

### High-strength HPF steels

A 1470-MPa-grade HPF steel was fabricated by a vacuum-induction melting route, and its chemical composition was Fe-(0.20–0.25)C-(0.2–0.3)Si-(1.1–1.2)Mn-(S + P + Ti + B + N) (wt%). The steel was homogenized at 1200 °C for 1 h, hot-rolled at 1100–900 °C, held at 600 °C for simulating a coiling procedure, cooled to room temperature, and cold-rolled to produce 1.5-mm-thick sheets. The steel sheets were annealed at 780 °C, coated by Zn or Al-Si at a decreased temperature under a reducing atmosphere, cooled to room temperature. The coated sheets were austenitized at 900 °C for 6 min and quenched into the water. The HPF steel sheets coated by Zn or Al-Si are referred to as ‘Z’ or ‘AS’, respectively. A baking treatment is commonly used for painting of automotive parts; thus, the Z sheet was additionally baked at 170 °C for 20 min to investigate its effect on bending behavior. The baked ‘Z’ sheet is referred to as ‘ZB’, for convenience.

### Microstructural characterization and tensile and bending tests

The sheets were mechanically polished and etched in a solution of (4 g picric acid + 4 g sodium dodecylbenzenesulfonate (SDBS) + 1 ml hydrochloric acid + 100 ml distilled water) at 100 °C to reveal prior austenite grain boundaries. Microstructures of longitudinal-short-transverse (L–S) plane were observed by using an optical microscope and a scanning electron microscope (SEM, JSM-7100F, JEOL, Japan, voltage; 10 kV). Constituent phases were examined by an X-ray diffraction (XRD, CuK_α_ radiation, Rigaku D/Max-2500V, Japan). Contents of elements such as C, Zn, Si, Al, and Fe were measured along the depth direction from the coating surface to the steel interior (maximum depth; about 50 μm) by using a glow discharge spectrometer (GDS, GDS850A, Leco, USA).

Plate-shaped bar specimens (gage length; 25 mm, width; 6 mm, thickness; 1.4 mm, longitudinal orientation) were tensioned at a strain rate of 10^–3^ s^−1^ by using a servo-hydraulic testing machine (8801, Instron, USA, capacity; 100 kN) at room temperature. Vickers hardness was measured under a 100 g load. All tests were carried out three times to ensure the data reliability.

**Figure 11:**
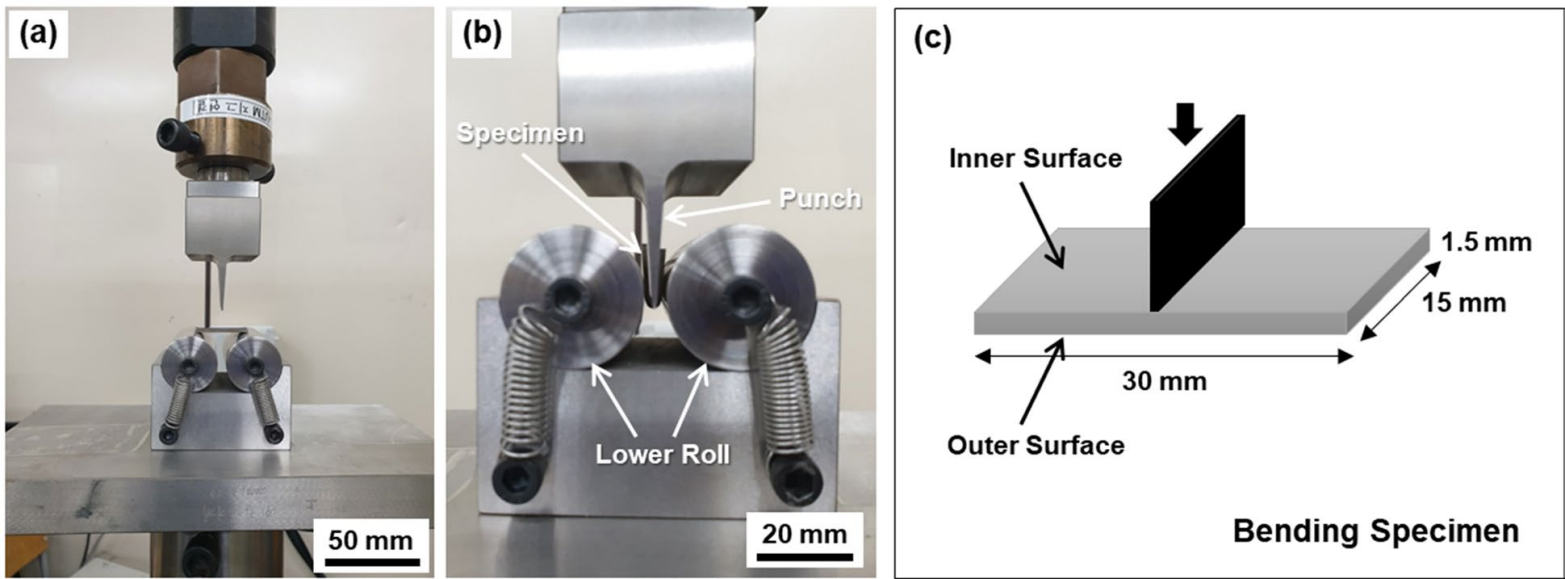
(**a**,**b**) Photographs of the three-point bending test set-up composed of punch, lower rolls, and bending specimen and (**c**) shape and dimensions of test specimen.

Three-point bending tests were conducted on sub-sized rectangular sheets (30 × 15 × 1.5 mm, longitudinal orientation) at a crosshead speed of 0.02 mm/s in accordance to the VDA 238–100 standard^34^ by using the servo-hydraulic testing machine. Figure 11a–c shows the bending test set-up composed of punch, lower rolls, and bending specimen. Interrupted bending tests were also conducted on the same-sized specimens at an interval of a bending angle of 3°. According to the previous bending procedures coupled with a digital image correlation (DIC) technique^35–37^, the local tensile strain was concentrated mainly at the center of the outer surface of the bend specimen. Thus, the specimen was bent at a certain bending angle, taken out from the test set-up, and half-sectioned along the longitudinal direction. The reason for sectioning was because the observation of the specimen surface (L–S plane) was not appropriate for examining the bending procedures due to the formation of heavily deformed shear bands on the surface. The central area of the outer surface was polished, etched in a 1%-nital solution, and observed by an SEM.

## Data Availability

The data that support the findings of this study are available from the corresponding author upon reasonable request.
